# The FXII c.-4T>C Polymorphism as a Disease Modifier in Patients With Hereditary Angioedema Due to the FXII p.Thr328Lys Variant

**DOI:** 10.3389/fgene.2020.01033

**Published:** 2020-09-10

**Authors:** Fernando Corvillo, María Eugenia de la Morena-Barrio, Carmen Marcos-Bravo, Margarita López-Trascasa, Vicente Vicente, Jonas Emsley, Teresa Caballero, Javier Corral, Alberto López-Lera

**Affiliations:** ^1^Centre for Biomedical Network Research on Rare Diseases (CIBERER), Madrid, Spain; ^2^Hospital La Paz Institute for Health Research (IdiPaz), Madrid, Spain; ^3^Hematology and Medical Oncology Department, University Hospital Morales Meseguer, Centro Regional de Hemodonación, University of Murcia, IMIB-Arrixaca, Murcia, Spain; ^4^Allergy Department, University Hospital Complex of Vigo, Hospital Meixoeiro, Vigo, Spain; ^5^Faculty of Medicine, Autonomous University of Madrid, Madrid, Spain; ^6^Centre for Biomolecular Sciences, School of Pharmacy, University of Nottingham, Nottingham, United Kingdom; ^7^Allergy Department, La Paz University Hospital, Madrid, Spain

**Keywords:** hereditary angioedema, hereditary angioedema with normal C1-Inhibitor, hereditary angioedema due to FXII mutations, *F12* gene, genetic disease-modifier

## Abstract

**Background:**

Hereditary angioedema due to the Thr328Lys variant in the coagulation factor XII (HAE-FXII) affects mainly women in whom the symptomatology is dependent on high estrogen levels. Clinical variability and incomplete penetrance are challenging features that hinder the diagnosis and management of HAE-FXII. The c.-4T>C Kozak polymorphism is the only common variation accounting for FXII plasma levels and was previously shown to modify the course of HAE due to C1-Inhibitor deficiency.

**Objectives:**

To assess the influence of the c.-4T>C polymorphism on disease expression in 39 Spanish HAE-FXII index patients.

**Methods:**

The c.-4T>C polymorphism was sequenced by the standard Sanger method, and HAE severity was calculated according to the score by Cumming et al. (2003) The activation of the contact system was quantified by the kallikrein-like activity of plasma in chromogenic assays upon activation with high-molecular-weight dextran sulfate.

**Results:**

The c.-4CC genotype was overrepresented in the studied cohort: 82% were CC-homozygous (expected frequency = 59%) and 18% were CT-heterozygous (expected frequency = 39%) (*p* = 0.001). Patients with a c.-4CC genotype exhibited higher kallikrein-like activity (0.9659 ± 0.1136) than those with a c.-4TC genotype (0.7645 ± 0.1235) (*p* = 0.024) or healthy donors. Moreover, the polymorphism influenced HAE-FXII severity score (c.-4CC = 4.43 ± 2.28 vs c.-4TC = 2.0 ± 1.15; *p* = 0.006) but not the degree of estrogen dependence or time until remission.

**Conclusion:**

The c.-4T>C polymorphism is overrepresented in a Spanish HAE-FXII cohort and significantly influences the degree of contact system activation and the clinical severity of the disease.

## Introduction

FXII or Hageman factor is the zymogen of the protease FXIIa and an emerging molecule in hemostasia and thromboinflammation (Ratnoff and Colopy, 1955). Despite the fact that FXII was originally described as a member of the intrinsic pathway of coagulation, FXII-deficient patients have nevertheless a normal hemostatic capacity and do not suffer from spontaneous or injury-related increased bleeding (mild to absent bleeding diathesis) but are indeed protected from thromboembolic diseases (Zeerleder et al., 1999; Colman, 2001; Kleinschnitz et al., 2006). Despite its physiological role remaining elusive for decades, it is now understood that besides its function in the intrinsic pathway of coagulation, which is dispensable for coagulation *in vivo*, FXII is implicated in an array of other biological phenomena from fibrinolysis to neutrophil recruitment (Björkqvist et al., 2015).

The plasma contact (or kallikrein-kinin) system consists of the coagulation factors FXI and FXII, the zymogen plasma prekallikrein (PK), and the cofactor and enzymatic substrate high-molecular-weight kininogen (HK). Upon contact with either artificial (glass, silica, dextran sulfate…) or physiological (polyphosphates, endotoxin, amyloid protein, heparins.) activators, FXII autoactivates by limited proteolysis to FXIIa and cleaves PK to its active form (PKa) which for its part promotes further activation of PK and FXII molecules in a reciprocal manner. Locally produced FXIIa initiates the intrinsic pathway of coagulation via its substrate FXI and leads to the release of the proinflammatory mediator bradykinin (BK) by PKa-mediated cleavage of HK (Hofman et al., 2016).

Uncontrolled activation of the plasma contact system is on the basis of the rare, life-threatening disease hereditary angioedema (HAE). HAE can arise from loss-of-function variants in the *SERPING1* gene encoding C1-Inhibitor (HAE-C1INH) or alternatively present with normal levels and function of C1INH (HAE-nC1INH). Among HAE-nC1INH patients, a proportion of cases bear heterozygous variants in the *F12, PLG, ANGPT1, or KNG1* genes resulting in HAE-FXII, HAE-PLG, HAE-ANGPT1, and HAE-KNG1 disease variants, respectively (Maas and López-Lera, 2019). In the remaining patients with HAE-nC1INH, the genetic defect responsible for the phenotype is not yet known. Plasma samples from patients with HAE exhibit impaired control over the plasma contact system resulting in its enhanced activation (Bova et al., 2019).

The c.1032C>A (p.Thr328Lys, rs118204456) pathogenic variant in the coagulation FXII is the most frequent cause of HAE-FXII. Haplotype analyses have confirmed that p.Thr328Lys is a founder-effect variant probably originating from Centro-European population around the XIth century (Cichon et al., 2006; Firinu et al., 2015). Mounting evidence on the physiopathology of HAE suggests that p.Thr328Lys has a profound effect on the protein’s folding and conformation. FXII molecules with the variant adopt an open or relaxed conformation which facilitates the access of PKa and plasmin to their FXII-activating moieties and also exposes cryptic proteolytic targets for thrombin which are normally concealed and not accessible in the compact conformation of FXII (de Maat and Maas, 2016). Moreover, the cleavage of FXII^Thr 328Lys^ by thrombin leads to enhanced activation of the contact system and bradykinin-mediated angioedema (Ivanov et al., 2019).

The p.Thr328Lys variant thus renders FXII susceptible to make exacerbated responses to both conventional (PKa) and alternative (thrombin) activators, which entitles it as either a neomorphic or hypermorfic allele (Muller, 1932). In this regard, the acquisition of this novel interaction between FXII^Thr328Lys^ and thrombin can be considered as a moonlighting activity bridging two otherwise physiologically separated systems. Thus, this interaction results in a pathological phenotype that is qualitatively different from those previously associated with FXII mutations. Limited information exists to date as to how p.Thr328Lys or wild type gene dosages modify the phenotype in HAE-FXII patients. Data from two Brazilian HAE-FXII patients (symptomatic male and female) bearing the p.Thr328Lys variant in homozygous state who manifested more severe HAE phenotypes suggest that it is indeed a hypermorphic variant (Grumach et al., 2016).

The c.-4T>C polymorphism (also referred to as *F12*-46C/T; rs1801020) in the 5′-UTR region of the *F12* gene potentially affects its mRNA translation levels by two independent mechanisms (Kanaji et al., 1998). First, the T allele disrupts a canonical Kozak sequence [GCC(A/G)CCAUGG], which precludes a normal recognition of the translation initiation signal. Second, it introduces a novel ATG codon in close proximity to the canonical one that translates a short ORF (9 bp) which prevents the normal elongation of nascent mRNAs probably leading to ribosomal arrest and poor translation efficiency. Due to its detrimental effect on translation efficiency, c.-4T>C has been proved to act as a genetic disease modifier by influencing FXII and FXIIa plasma levels in hemostatic pathology and coronary artery disease (Ardissino et al., 1999; Kohler et al., 1999; Ishii et al., 2000; Endler et al., 2001b; Firinu et al., 2015; Roldán et al., 2005; Bach et al., 2008; Corral et al., 2010).

The genotype distribution of the c.-4T>C polymorphism is strikingly diverse among different human subpopulations. While in Europeans the C allele is more represented [genotypes distribution: 60% (CC), 35% (TC), and 5% (TT)], a different distribution is observed in South Asians [30% (CC), 20% (TC), and 50% (TT)] and East Asians [(58% (CC), 5% (TC), and 37% (TT)]. In the Iberian subpopulation, genotype distribution is 59% (CC), 39% (TC), and 2% (TT) (The 1000 Genomes Project Consortium, Phase 3).

Previous reports have shown that c.-4T>C has also a considerable impact on the course of HAE-C1INH. In a study involving 152 European patients, those carriers of the T allele were shown to exhibit a significantly delayed disease onset and did not need long-term treatment (Speletas et al., 2015). Accordingly, a recent study on southeastern European population has demonstrated that the C allele is overrepresented in HAE-C1INH patients while the T allele is associated to an asymptomatic course of the disease (Rijavec et al., 2019). Of note, haplotype analysis by Cichon et al. (2006) and Firinu et al. (2015) has shown that the c.1032C>A (p.Thr328Lys) variant originated in an extended haplotype presenting a C allele at c.-4 position and is therefore a high-expression allele.

In the present report, we investigated whether the c.-4T>C genotype of the complementary c.1032C (p.328Thr) wild-type allele present in heterozygous HAE-FXII patients can modulate the severity of symptoms and the activation status of the plasma contact system during remission.

## Results

Thirty-nine Spanish women of child-bearing age with an established diagnosis of HAE-FXII due to the p.Thr328Lys (c.1032C>A) variant were genotyped for their c.-4T>C status. The clinical data of these patients were collected and validated by their referring clinicians (Table 1). All of them presented conserved levels and function of C1INH and C4 at the time of plasma extraction.

**TABLE 1:** HAE-FXII cohort.

**Patient code**	**Kozak genotype**	HAE clinical score*	**Strict estrogen dependence**	**Time to resolution of HAE attacks (hours)**
1	CC	1	1	96
2	CC	7	1	ND
3	CC	2	1	72
4	CC	2	1	72
5	CC	5	0	120
6	CC	6	1	ND
7	CC	4	1	ND
8	CC	ND	1	96
9	CC	2	1	96
10	TC	4	1	24
11	CC	5	0	60
12	CC	3	0	72
13	CC	6	1	18
14	TC	1	1	60
15	TC	1	1	24
16	CC	2	0	84
17	CC	3	1	96
18	CC	7	0	72
19	CC	3	1	96
20	CC	2	1	36
21	TC	1	0	72
22	TC	3	0	ND
23	CC	7	1	48
24	CC	7	1	80
25	CC	8	0	72
26	CC	2	1	48
27	CC	8	1	48
28	CC	4	1	72
29	CC	8	0	48
30	CC	6	1	36
31	CC	7	1	48
32	CC	4	0	ND
33	CC	5	1	ND
34	CC	ND	1	ND
35	TC	2	0	96
36	TC	2	0	96
37	CC	2	1	ND
38	CC	4	ND	24
39	CC	1	1	96

Thirty-nine female index patients with HAE-FXII due to the heterozygous c.1032C>A (p.Thr328Lys, rs118204456) variant were genotyped for their c.-4T>C (rs1801020, Kozak polymorphism) status. Clinical records obtained at the time of diagnosis were used to calculate their HAE clinical score (0–10; *see Supplementary Table S1), strict dependence of HAE symptoms on estrogen levels (1: estrogen-dependent/0: estrogen-sensitive) and time required until the spontaneous (i.e., in the absence of specific treatments) remission of symptoms (hours). ND, not determined.

**FIGURE 1:**
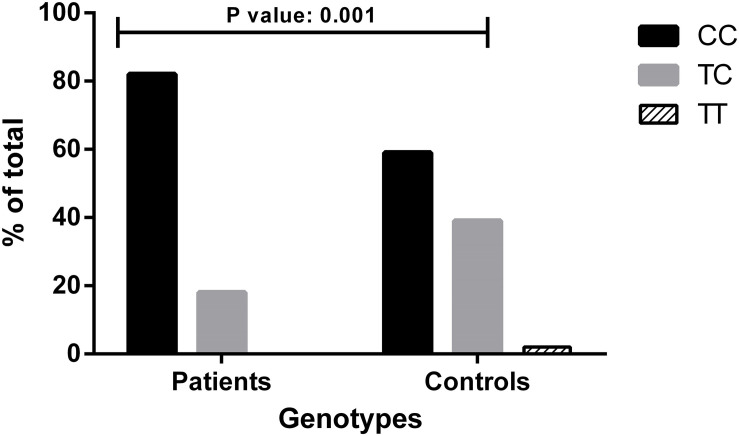
Genotype distributions of the c.-4T>C (rs1801020) polymorphism in patients with HAE-FXII and healthy donors from the Iberian population. Genotype distribution for Iberian population was drawn from The 1000 Genomes Project Consortium, Phase 3. Statistical analysis was performed with Fisher’s exact test. Data are statistically significant for *p*-values ≤ 0.05.

In the studied population, the genotype distribution of the c.-4T>C polymorphic variant was consistent with the Hardy-Weinberg equilibrium (χ^2^ *p*-value: 0.2407). Among the 39 patients, 32 were CC homozygous, and 7 were CT heterozygous for c.-4T>C. No TT homozygous were present in the cohort, in line with the fact that the haplotype linked to the p.Thr328Lys (c.1032C>A) variant contains a C at position c.-4 (Cichon et al., 2006; Firinu et al., 2015). To avoid stratification bias due to the different population-specific allele frequencies, we compared the genotype distribution seen in HAE-FXII patients bearing the p.Thr328Lys variant with that expected for Iberian population and found a significant overrepresentation of the C allele in the patient group (Fisher exact test—*p*-value: 0.001) (Figure 1).

We based this investigation on the hypotheses that (i) c.-4T>C acts as a disease modifier in HAE-FXII patients and (ii) this in its turn influences the penetrance of the p.Thr328Lys variant resulting in a diagnostic bias in HAE-FXII cohorts and in the overrepresentation of the c.-4CC genotype. In this regard, the enrichment in C alleles with respect to its expected frequency in the Spanish population is in agreement with the above hypotheses.

**FIGURE 2:**
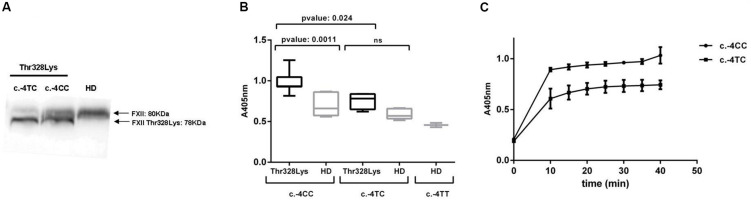
FXII levels and activation of the plasma contact system. **(A)** Western blot of plasma samples with polyclonal anti-human FXII showing the impact of the c.-4T>C genotype on the expression levels of the wild type (80 KDa) and variant (78 KDa) FXII variants bands. **(B)** The activation of the contact system was evaluated by measuring the kallikrein-like activity with the S2302 chromogenic substrate (*A*_405 nm_) upon DXS triggers on citrated plasma samples from HAE-FXII patients with the p.Thr328Lys variant (with either c.-4TC- or c.-4CC genotypes) and genotype-, age-, and sex-matched healthy donors (HD) from the Iberian population. S2302 end-point values are shown. **(C)** Time-course absorbance values at 405 nm obtained in two representative HAE-FXII patients with c.-4CC and c.-4TC genotypes (the results from duplicated samples are shown). Statistical analyses were performed with Mann–Whitney *U*-test. Data is statistically significant for *p*-values ≤ 0.05.

To better characterize the consequences of the polymorphism frequencies observed in our cohort, we aimed to investigate the impact of c.-4T>C on biochemical parameters relevant to HAE. It is well established in the literature that c.-4T>C determines the antigenic levels of both FXII (Figure 2A) and its active enzyme FXIIa (Kanaji et al., 1998). Therefore, we wondered whether it also influences the activation status of the contact system in HAE-FXII patients with the p.Thr328Lys variant during remission. The activation of the contact system was assayed in freshly thawed citrated plasma samples by short incubations with high-molecular-weight dextran sulfate (DXS) at different concentrations ranging from pg/mL to mg/mL. In order to allow for comparisons with healthy donors samples, a DXS working solution of 100 μg/mL was selected at which both healthy donors and patients exhibited measurable levels of S2302 signal.

As shown in Figure 2B, HAE-FXII patients with a c.-4CC genotype exhibit significantly higher kallikrein-like activity values [0.9659 ± 0.1136 (0.930–1.029, 95% CI)] than those with a c.-4TC genotype [0.7645 ± 0.1235 (0.4578–1.071; 95% CI)] and their corresponding age- and sex-matched healthy donors with either c.-4CC (0.6978 ± 0.1361; 0.5559–0.8396; 95% CI) or c.-4TC genotypes [0.5863 ± 0.07 (0.5222–0.6505; 95% CI)]. The kinetics of the S2302 reaction in two representative HAE-FXII patients with CC and TC c.-4T>C genotypes is shown in Figure 2C.

**FIGURE 3:**
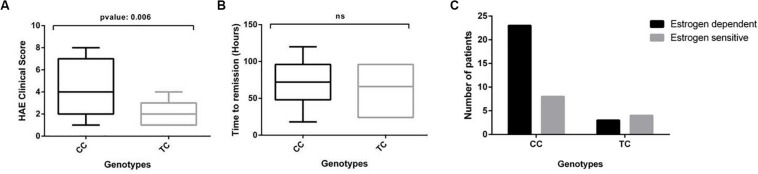
The clinical course of HAE is modified by the c.-4T>C genotype in HAE-FXII patients with the P.Thr328Lys mutation. **(A)** The c.-4CC genotype is significantly associated with higher HAE clinical scores in HAE-FXII patients with the P.Thr328Lys variant as compared to those with a TC genotype (Mann–Whitney *U*-test). **(B)** No significant effect was detected on the time required to reach complete remission of symptoms (Mann–Whitney *U*-test). **(C)** The frequency distribution of estrogen-dependent vs estrogen-sensitive patients according to their c.-4T>C genotype suggests clustering of estrogen-dependent cases into the c.-4CC genotype group. No contingency analysis could be performed on these data due to the small number of cases in the TC group (*n* < 5).

Kallikrein-like activity as measured with the S2302 substrate in our assay reflects overall activation of the contact system in plasma, which is on the pathological basis of HAE-FXII. Consequently, an increased kallikrein-like activity would in principle be expected to associate with a worse course of the disease. We therefore studied the HAE severity score and the time until remission of symptoms in the cohort and their distribution according to the c.-4T>C genotype of the patients in the absence of treatment (Figure 3).

HAE-FXII patients with a c.-4CC genotype exhibit more severe and frequent manifestations of the disease [SC: 4.43 ± 2.28 (3.58 – 5.28 95% CI)] than those with a c.-4TC genotype [SC: 2.0 ± 1.15 (0.93 – 3.06 95% CI)], which in turn led to significant differences (*p*-value: 0.006) in their average SC values (Figure 3A). No differences were observed in the time required until the spontaneous and complete remission of symptoms between both groups [mean: 68.24 h (c.-4CC) vs 62.00 h. (c.-4TC)] (Figure 3B). We also examined the dependency of symptoms with estrogens. Although the data suggest an effective clustering of estrogen-dependent cases into the c.-4CC genotype group, no statistical analyses could be performed due to the small number (*n* < 5) of cases with a TC genotype (Figure 3C).

Review of the clinical records showed differences in the predominant anatomical distribution of edema episodes in HAE-FXII patients depending on their c.-4T>C genotypes. In general, the milder peripheral skin affectation was reported by most of the patients (*n* = 18), and this was the main localization of edema in both groups, although it was striking majority in those with a c.-4TC [genotype: 12/30 with c.-4CC (40%) vs 6/7 with c.-4TC (86%)]. In contrast, a higher occurrence of more severe abdominal episodes (*n* = 11) was observed among patients with c.-4CC genotype: 10/30 with c.-4CC (33%) vs 1/7 with c.-4TC (14%). Of note, none of the seven c.-4TC patients reported angioedema episodes affecting the upper respiratory tract, while all eight patients showing the most severe clinical phenotype of upper airways episodes (n = 8) carried the c.-4CC genotype (Table 1).

The majority of the patients included experienced their first manifestations of the disease during the second decade of life coinciding with either their first pregnancy or the start of contraceptive therapies. However, considering the modifying effect of estrogens on the expressivity of HAE-FXII after puberty and the evident bias associated with this fact, we did not analyze the age at onset of symptoms.

## Discussion

Clinical variability and incomplete penetrance are still challenging characteristics for the diagnosis of the different HAE variant forms recognized to date. Although a major influence of environmental factors is evident (as exemplified by estrogen exposure in the case of HAE-FXII patients), part of the variability can also be ascribed to disease-modifying genes (Margaglione et al., 2019). Most of the HAE disease-modifiers identified are related with changes in the plasma activity of bradykinin catabolic enzymes. For example, the levels of angiotensin-converting enzyme (ACE) are largely influenced by the rare insertion/deletion polymorphism rs1799752 and in turn ACE activity in plasma inversely correlates with the severity of HAE-FXII (Rigat et al., 1990; Charignon et al., 2014). Moreover, bradykinin-mediated HAE is a genetically complex disease and patients with combined pathogenic variants in the *SERPING1* and *F12* genes have been described (Charignon et al., 2018) which further complicate management. In this regard, genotyping the c.-4T>C status may provide important information in order to help predict the severity of HAE, and moreover, it could be readily included in next generation sequencing platforms intended for HAE diagnosis. With regard to the *F12* gene, the c.-4T>C polymorphism is recognized as the only common variation accounting for the variability in the plasma levels of FXII (Calafell et al., 2010; Sabater-Lleal et al., 2010). Carriers of the T allele showed a significant reduction in the plasma levels of FXII that reach in TT homozygous half of the values observed in CC homozygous (Kanaji et al., 1998; Corral et al., 2010). Moreover, the high allele frequencies of this polymorphism in the general population (present in up to 35% of the European Caucasian healthy population) make it an approachable goal for clinical-association studies in relatively small cohorts.

From a phylogenetic perspective, there is evidence that c.-4T>C appeared at least twice during human evolution. It is a relatively ancient polymorphism that has been ubiquitously present in humans for at least 100,000 years and whose geographical spread can be fairly explained solely on the basis of genetic drift and the demographic history of humans (Calafell et al., 2010). All considered, c.-4T>C seems to have no significant detrimental effect on the individual’s reproductive fitness and can be considered as neutral in the general population. However, the situation might be different in HAE-FXII patients bearing the p.Thr328Lys in which FXII levels can influence the clinical expression of HAE and in turn (specially in ancient times) reduce their life-expectancy and reproductive fitness.

HAE-FXII pathology is primarily driven by an autosomal dominant FXII variant that over-activates and in turn induces the activation of further mutant and wild-type FXII molecules. Due to this infective-like behavior of the mutant FXII molecule, the expression levels of both the mutant and wild-type *F12* alleles probably have a significant influence on the overall activation status of the contact system.

Our results confirm the latter hypothesis in a small cohort of HAE-FXII index patients. On one hand, we found a significant over-representation of the C allele and the CC genotype in the studied patients (Figure 1), which is in line with previous studies implicating c.-4T>C in HAE-C1INH. In European HAE-C1INH cohorts, the c.-4TC genotype is associated with a delayed disease onset and a lesser need for long-term prophylaxis (Bors et al., 2013; Gianni et al., 2017; Liu et al., 2019). Similarly, studies performed by Rijavec and colleagues have found a significant association of the c.-4T allele with an asymptomatic phenotype in HAE-C1INH (Rijavec et al., 2019).

On the other hand, plasma from HAE-FXII patients with a c.-4CC genotype exhibited higher kallikrein-like activity (with the S2302 chromogenic substrate) upon low-dose DXS triggers than those with a c.-4TC genotype (Figure 2). Considering that PK is secondarily activated by FXIIa during contact system activation, this is in agreement with the initial reports about the influence of the polymorphism on the levels and function of FXII (Kanaji et al., 1998). Moreover, increased S2302 signals in those HAE-FXII patients with a c.-4CC genotype were accompanied by a more severe presentation of the disease as compared to c.-4TC cases (severity scores 4.43 vs 2.00) (Figure 3A) but did not influence the time required to reach the complete remission of attack symptoms (Figure 3B). These data suggest that therapies targeting FXII expression which have been proposed for patients with HAE-C1INH (Liu et al., 2019) may also be considered for HAE-FXII patients. Further studies investigating the segregation of the c.-4T>C and c.1032C>A alleles in asymptomatic relatives of the studied index patients may also supply new clues of the modulating effect of the functional polymorphism on the clinical consequences of the pathological variant.

By restricting the study to plasma samples taken only from index patients at the time of their diagnosis, we tried to obtain an unbiased estimation of the effect of the polymorphism on the expression of the disease and its diagnostic rates. The inclusion of index-patients’ relatives receiving genetic counseling once the disease has been recognized within their families would probably incorporate yet asymptomatic or less-severely affected individuals, leading to an overestimation of the diagnostic rates.

Although only women with HAE-FXII were included in the present study, we speculate that our findings might also be applicable to male patients. HAE-FXII is predominantly detected in women but it is now assumed that men can also be affected and cases of male-to-male transmission of the disease have been documented. Affected males with the p.Thr328Lys variant are rare and usually present with less severe and less frequent symptoms, delayed onset of the disease and kallikrein-like activity values far below those seen in women patients (Martin et al., 2007). Estrogen responsiveness of FXII due to ERα receptor binding to cDNA positions −43/−31 of the *F12* promoter is a transcriptional phenomenon, while the c.-4T/C variant modifies FXII synthesis at the translational level by modulating the translation efficiency of mRNA molecules (Kanaji et al., 1998). No evidence of interaction exists between these processes in the *F12* gene, and therefore we hypothesize that the c.-4T/C variant probably acts independently of the patients’ gender or hormonal balance.

In conclusion, the results presented here indicate that c.-4T>C acts as a genetic disease modifier partially determining the degree of contact system activation and the clinical severity of HAE-FXII patients with the p.Thr328Lys variant. Moreover, in consideration of its clinical impact and overrepresentation in the studied cohort, c.-4T>C in turn possibly influences the diagnostic rates of the disease. In line with this, it is not dismissing to suggest that the recognition of a proportion of HAE-FXII cases in different populations might be hindered by a masking effect on their symptoms due to the presence of the c.-4T allele segregating at high frequencies.

## Materials and Methods

### Patients, Biological Samples, and Clinical Data

Thirty-nine Spanish women of child-bearing age with an established diagnosis of HAE-FXII due to the p.Thr328Lys (c.1032C>A) variant in heterozygosis were recruited for these studies between 2009 and 2017 at Hospital Universitario La Paz. Those included were all normocomplementemic women index patients above 18 years old with HAE symptoms. In line with generally accepted criteria (Bouillet, 2010), HAE symptoms were considered as “estrogen-dependent” in those patients presenting angioedema attacks exclusively during periods characterized by high levels of estrogens (oral contraceptive intake, menstruation, pregnancy…). Conversely, symptoms were classified as “estrogen-sensitive” in those patients in whom the symptoms are worsened by high circulating levels of estrogens. Whole blood and citrated plasma samples were obtained during asymptomatic, remission periods. Additionally, samples from forty healthy donors with no analytical or clinical evidence of angioedema were obtained from the Immunology Unit of Hospital La Paz. The Ethics Review Panel of La Paz University Hospital reviewed and approved this study meeting the requirements of the 2013 Helsinki declaration and later amendments. All patients and healthy donors included were adequately informed and gave written consent to participate.

### DNA and RNA Studies

DNA and RNA samples were obtained from peripheral blood mononuclear cells using Gentra Puregene Blood Core and RNeasy Midi kits from Qiagen (Valencia, CA, United States), respectively, following the manufacturer’s instructions. The *F12* gene was amplified by polymerase chain reaction with either DNA- or RNA-specific primers and sequenced as described previously (Marcos et al., 2012). Throughout the document, the rs1801020 c.-4T>C Kozak polymorphism is named c.-4T>C and the rs118204456 missense variant as c.1032C>A (p.Thr328Lys) according to the reference transcript ENST00000253496.3.

### Kallikrein-Like Activity in Plasma

The activation of the plasma contact system was evaluated as the kallikrein-like activity measured in citrated plasma samples following the protocols by Kluft (1978) and Defendi et al. (2013) with slight modifications. Briefly, 96-well Costar Medium Binding plates (Corning; Madrid, Spain) were coated with 50 μL DXS at 100 μg/mL in PBS overnight at 4°C. Plates were then saturated with 50 μL of 1 mg/mL skimmed milk in PBS for 1 h at room temperature, and 20 μL freshly thawed citrated plasma samples were added in duplicates. After a 10-min incubation at 4°C, the chromogenic substrate H-D-Pro-Phe-Arg-pNA (S2302; Chromogenix, Milan, Italy) (0.5 mM final concentration) was added, and pNA release was monitored for 1 h at room temperature with a microplate reader at 405 nm.

### Western Blot

Plasma proteins were separated by 4–20% gradient SDS-PAGE gels (Bio-Rad, Madrid, Spain) under reducing conditions and blotted on a polyvinylidene difluoride membranes. FXII was immunostained with a polyclonal anti-human FXII antibody developed in goat (GAFXII-AP; Enzyme Research Laboratories, South Bend, IN, United States), followed by a monoclonal anti-goat IgG-HRP linked antibody (A9452-1VL, Sigma-Aldrich, Madrid, Spain). Detection was done with chemiluminescence (ECL^TM^ Prime; Amersham).

### Clinical Score

The severity of HAE symptoms was measured according to a modified version of the score introduced by Cumming et al. (2003) based on the location and frequency of attacks. Cumming et al. distinguished those asymptomatic patients presenting with conserved complement levels (score = 0) from those without symptoms but with hypocomplementemia (score = 0.5). Considering that complement levels are conserved in HAE-FXII patients during relapse, asymptomatic patients in our series were assigned a clinical score of 0 (Supplementary Table S1). The age at onset of symptoms was not taken into consideration because of the significant environmental influence of estrogens levels on this parameter in HAE-FXII.

### Statistical Analysis

GraphPad Prism 7 software (GraphPad Software, La Jolla, CA, United States) was used for data analysis and graphics design.

## Data Availability Statement

All datasets presented in this study are included in the article/Supplementary Material.

## Ethics Statement

The studies involving human participants were reviewed and approved by Comité de Ética para la Investigación Clínica del Hospital Universitario La Paz. The patients/participants provided their written informed consent to participate in this study.

## Author Contributions

FC and AL-L performed the experimental work. AL-L, MM-B, and JC conceived the study and wrote the manuscript with support from JE and VV. TC, CM-B, and ML-T provided and managed the clinical data. All authors contributed to the article and approved the submitted version.

## Conflict of Interest

The authors declare that the research was conducted in the absence of any commercial or financial relationships that could be construed as a potential conflict of interest.

## Supplementary Material

TABLE S1 Severity scores for HAE-FXII patients. The severity of HAE symptoms was measured taking into account the location and frequency of edema episodes in the absence of treatment and according to a modified version of the score introduced by Cumming et al. (2003). The course of HAE was considered “severe” when a history of laryngeal edema is referred; intermediate course denotes the presence of gastrointestinal symptoms of HAE requiring hospitalization and/or specific medication; “mild” course implies the presence of HAE symptoms with neither the need for hospitalization nor specific medication. (‡) = death.

## Abbreviations

HAE: hereditary angioedema
HAE-FXII: hereditary angioedema due to FXII mutations.
