# Supplementary material for: The FXII c.-4T>C Polymorphism as a Disease Modifier in Patients With Hereditary Angioedema Due to the FXII p.Thr328Lys Variant
Source: Front Genet. 2020 Sep 10;11:1033. doi: 10.3389/fgene.2020.01033 (PMC7549737; doi:10.3389/fgene.2020.01033)
Supplement: TABLE S1 — Severity scores for HAE-FXII patients. The severity of HAE symptoms was measured taking into account the location and frequency of edema episodes in the absence of treatment and according to a modified version of the score introduced by Cumming et al. (2003). The course of HAE was considered “severe” when a history of laryngeal edema is referred; intermediate course denotes the presence of gastrointestinal symptoms of HAE requiring hospitalization and/or specific medication; “mild” course implies the presence of HAE symptoms with neither the need for hospitalization nor specific medication. (‡) = death. [file Data_Sheet_1.PDF]

Phenotype	Course of disease	Frequency of attacks per year	Score
**Fatal**	Fatal (‡)		**10**
**Severe**	Edema of the upper respiratory tract	>24	**9**
		12-24	**8**
		<12	**7**
**Intermediate**	Gastrointestinal edema	>24	**6**
		12-24	**5**
		<12	**4**
**Mild**	Subcutaneous edema	>24	**3**
		12-24	**2**
		<12	**1**
**Asymptomatic**		**0**	**0**
